# Association of Body Size and Muscle Grip Strength With Hypertension: A Cross-Sectional Study

**DOI:** 10.7759/cureus.105126

**Published:** 2026-03-12

**Authors:** Pratyaksh Gurnani, Pranav Dighe, Ruchi Kothari, Manish Rathod, Shreyash Yedke, Priya Jain, Senthil Kumar, Prashanth A

**Affiliations:** 1 Physiology, Mahatma Gandhi Institute of Medical Sciences, Wardha, IND; 2 Pharmacology and Therapeutics, Mahatma Gandhi Institute of Medical Sciences, Wardha, IND; 3 Anaesthesia, Santokba Durlabhji Memorial Hospital, Jaipur, IND; 4 Physiology, Madha Medical College and Hospital, Chennai, IND

**Keywords:** hand grip strength, hypertension, muscle grip, waist circumference, waist-to-height ratio

## Abstract

Background

Hypertension is a major contributor to global cardiovascular morbidity and mortality. Emerging evidence suggests that reduced hand grip strength (HGS) might be linked to elevated blood pressure and adverse cardiometabolic outcomes. Anthropometric measures like body mass index (BMI) and waist-to-height ratio (WHtR) have been recognized as potential markers of cardiometabolic risk. However, the combined influence of body size parameters, muscle strength, and hypertension in the rural Indian population remains underexplored.

Objective

Aiming to fill the research gap, the study’s objective was to compare anthropometric parameters and handgrip strength between hypertensive and normotensive adults residing in rural central India and to evaluate their associations with hypertension.

Methods

This comparative, cross-sectional, analytical study was conducted at a rural medical college of central India. The study sample consisted of 100 participants divided into 50 cases (hypertensive) and 50 age- and sex-matched controls (normotensive). Standardized protocols were followed while measuring HGS and anthropometric data. Maximum and mean HGS assessed by the grip force transducer were compared between the groups. Blood pressure measurements were recorded before and after the handgrip exercise protocol. Differences between groups were analyzed using the independent samples Student’s t-test, and Pearson correlation analysis was performed to evaluate associations between HGS, WHtR, and blood pressure.

Results

The mean age of 50 controls was 48.90 ± 10.68 years, and that of 50 hypertensives was 49.33 ± 10.97 years. There was no significant difference in height and weight between hypertensive cases and normotensive controls. WHtR was found to be significantly higher in hypertensive individuals (0.587 ± 0.11 vs. 0.49 ± 0.10, p = 0.0067), suggesting an association between central adiposity and hypertension. Hypertensive participants showed significantly (p < 0.001) lower mean HGS (58.32 ± 14.19 N) and maximum HGS (215.26 ± 58.95 N) compared to normotensive controls (77.25 ± 20.64 N and 335.06 ± 113.22 N, respectively). A statistically significant, strong negative correlation (r = -0.32, p = 0.020) was seen between maximum HGS and WHtR, and a statistically significant positive relation (r = 0.24, p = 0.044) between maximum HGS and heart rate.

Conclusion

The study aimed to reveal the inter-relationships of body size parameters like WHtR, muscle grip strength, and hypertension. The patients had significantly lower mean HGS and maximum HGS values as compared to normotensive controls and a higher WHtR, which underpins the role of muscle function and central adiposity in cardiovascular risk assessment and screening.

## Introduction

Hypertension, characterized by elevated blood pressure (BP) in the arteries, remains one of the most prevalent non-communicable diseases worldwide, increasing the risk of cardiovascular diseases, various strokes, and renal failure, which accounts for nearly one-third of global annual deaths [1]. Despite the availability of effective pharmacological therapies, the prevalence of hypertension continues to rise, particularly in low and middle-income countries like India, and as close to 30% of Indian adults are hypertensive [2].

The disease has reached epidemic proportions, and developing countries are unduly impacted by its escalating burden, so early identification of individuals at increased risk becomes essential, particularly in low-resource rural settings.

Muscle strength plays an important role in glucose metabolism, vascular function, and neural regulation, all of which are key mechanisms involved in BP control [3,4]. Thus, it has been identified as a potential defensive factor against hypertension contributing to reduced arterial stiffness, increased vasodilation, and better metabolic health, collectively causing improved BP regulation [5-8].

Hand grip strength (HGS) serves as a vital functional metric in evaluating muscle performance and physical capability and is specifically pertinent to the aging population [9,10]. It upholds a crucial connection with the age-related reduction in muscle mass and has been extensively employed as a clinical index of reduced mobility [11,12].

A hallmark of essential hypertension is autonomic dysfunction, mainly manifested as exaggerated sympathetic activation with parasympathetic withdrawal [13]. It has been shown that HGS reflects the neuromuscular function, and its deterioration contributes to weakened muscle function [14]. It also serves as a reliable measure of neuromuscular integrity, hence its evaluation provides significant insight into the adequacy of autonomic nerve functionality [15]. Only a couple of studies [16,17] in the past have suggested a negative relation between muscle strength and adverse cardiovascular outcomes. Sayer et al. had proposed a graded association between weaker grip strength, a marker of sarcopenia, and metabolic syndrome [16]. Lee and Park [17] have postulated the role of myosteatosis, i.e., ectopic adipose infiltration in skeletal muscles, to cause their attenuation and increase the risk of hypertension. Despite such evidence, the extent to which grip strength mediates hypertension risk still remains unclear.

Central adiposity, commonly measured using the waist-to-height ratio (WHtR), is a well-established predictor of hypertension [18]. WHtR is increasingly recognized as a robust indicator of visceral adiposity because it reflects abdominal fat accumulation relative to body size. Central adipose tissue is metabolically active and releases inflammatory cytokines, adipokines, and free fatty acids that promote insulin resistance, endothelial dysfunction, and increased sympathetic activity, all of which contribute to elevated BP. Hence, it is a reliable and convenient metric for identifying individuals with increased cardio-metabolic risk [19].

This consideration is particularly important in Asian populations, including the Indian population, because here people tend to develop central obesity at lower BMI values. Since BMI does not account for fat distribution, it may underestimate cardiovascular risk in such a population. Thus, incorporating anthropometric indicators that better capture central fat distribution, such as WHtR and waist circumference, into risk assessment may improve early identification of persons at increased cardiometabolic risk, especially in settings where BMI alone may not adequately reflect visceral adiposity.

Although studies have indicated that WHtR is a superior index of cardiovascular risk than BMI, as it depends on the distribution of fat and is less influenced by age, sex, and ethnicity compared with BMI [20], its relationship with muscle strength and hypertension has not been examined concurrently.

Though it has been documented [18,19] that greater WHtR results in poor muscle quality and decreased mitochondrial efficiency, leading to diminished functional capacity and metabolic dysfunction, there is existing evidence of correlation of body size parameters and muscle grip strength in relation to hypertension that is inconsistent, and their interplay is not well understood till now, particularly in the rural Indian population.

Since the assessment of HGS with a "matrix" based on BMI, waist circumference, or WHtR for identifying cardiometabolic risk has not been addressed so far, this study aimed to compare body size parameters and handgrip strength between hypertensive and normotensive adults residing in rural central India and to evaluate their associations with BP parameters.

## Materials and methods

Study design and setting

This study was conducted as a comparative cross-sectional analytical design. Participants were recruited during the study period and assessed at a single time point for anthropometric measurements, HGS, and BP using standardized protocols. Participants were matched for age and sex to minimize the influence of major demographic determinants of HGS. Clinical conditions such as diabetes mellitus, chronic kidney disease, previous cardiovascular events, and antihypertensive medication burden were not systematically recorded for all participants.

The study was conducted at the sports physiology lab of a rural medical college located in central India during the period from 5th March 2023 to 5th December 2023.

Sample size

Sample size was calculated using OpenEpi software (version 3.01), employing the following formula for comparing two proportions in the case-control study:

\begin{equation}n = \frac{(Z_{\alpha/2} + Z_{\beta})^2 \times \left[ P_1(1 - P_1) + P_0(1 - P_0) \right]}{(P_1 - P_0)^2}\end{equation}

Considering the exposure prevalence of 30% [2], a 95% confidence level, 80% power, and a 1:1 case-control ratio, the calculated minimum sample size was 40 per group. After adjusting for a potential 20% non-response, the final sample size was kept as 50 cases and 50 controls.

Study population and selection criteria

A total of 100 subjects in the age group of 35-65 years were included in the study, which were categorized into two groups: hypertensive group, including 50 individuals previously diagnosed with hypertension, and normotensive group of 50 age- and sex-matched individuals without hypertension.

As per the 2020 International Society of Hypertension Global Hypertension Practice Guidelines, hypertension was said to be diagnosed when repeated BP measurements demonstrate a systolic BP of ≥140 mm Hg and/or a diastolic BP of ≥90 mm Hg [21]. Patients with physical disabilities affecting hand grip strength, chronic illnesses such as ischemic heart disease, diabetes, and chronic obstructive pulmonary disease, and who refused to give consent were excluded from the study.

Ethics consideration

The research project was duly approved by the Institutional Ethics Committee (Approval No.: MGIMS/IEC/ANAT/36/2019; dated: 09/03/2019). A signed written informed consent was obtained from all study participants prior to enrollment. The consent was fully informed, and was given voluntarily by all the participants. Each subject had been notified of the risks, benefits, and possible discomforts of the study.

Data sources and measurement of variables

Anthropometric Measurements

Height was recorded in centimeters and weight in kilograms (bare feet) on the Phoenix Height Weight Body Mass Index machine (Model: PBMI-200; Nitiraj Engineers Ltd., Dhule, India). The BMI automatically displayed on the screen was noted. Waist circumference was measured at the midpoint between the iliac crest and the lower rib using a non-stretchable measuring tape. WHtR was calculated as waist circumference divided by height.

Measurement of Handgrip Strength

HGS was assessed using a grip force transducer following a standardized protocol [22]. Mean HGS was calculated as the average force generated over a two-minute period. The recording and analysis of results were done using LabChart Pro software (ADInstruments, New Delhi, India), which provides a display of all the necessary data in a graphical manner and calculates all the requisite parameters for analysis while the subject is performing the test.

Blood Pressure Measurement

BP was measured using a mercury-free BP apparatus (LED-Regular BPDG141, Diamond, Pune, India). Readings were taken and noted before and after the HGS test. BP measurements in the present study were obtained using clinic-based readings. Although contemporary guidelines define hypertension at a lower threshold of ≥130/80 mmHg, the conventional cut-off considered in the study is consistent with epidemiological studies, particularly in resource-limited settings of rural central India, where 24-hour ambulatory or home monitoring was not available for routine use in the study population during data collection.

Statistical analysis

Data analysis was performed using IBM SPSS Statistics for Windows, version 25 (IBM Corp., Armonk, NY). Descriptive statistics were expressed as mean ± standard deviation (SD). Differences between hypertensive and normotensive groups were analyzed using the independent samples Student’s t-test. Pearson’s correlation coefficient was used to assess relationships between anthropometric variables, HGS, and BP parameters. A significance level of p < 0.05 was considered statistically significant.

## Results

The mean age of 50 controls was 48.90 ± 10.68 years, and that of 50 hypertensives was 49.33 ± 10.97 years. Table 1 presents the demographic and clinical characteristics of study participants. The mean height of controls was 164.56 cm, and that of cases was 162.53 cm. The mean weight for normotensive controls was 59.31 ± 11.71 kg, while for hypertensive cases, it was 61.93 ± 7.37 kg. There was no significant difference in height, weight, and BMI between hypertensive cases and normotensive controls. The average waist circumference of cases was 95.33 cm, and that of controls was 81.22 cm. The average resting heart rate (HR) of hypertensives was 82.13, and that of controls was 78.03. WHtR was found to be significantly higher in hypertensive individuals (0.587 ± 0.11 vs. 0.49 ± 0.10, p = 0.0067), suggesting an association between central adiposity and hypertension. The mean systolic BP of hypertensive subjects before and after exercise was 130 mmHg and 138 mmHg, respectively, and that of controls was 116 mmHg and 122 mmHg, respectively. BP before exercise was significantly higher in the hypertensive cases (124 ± 18.27 mmHg) compared to controls (116 ± 8.41 mmHg, p = 0.049). Post-exercise BP remained elevated in hypertensive individuals (132 ± 21.25 mmHg vs. 122 ± 11.39 mmHg in controls, p = 0.045). Hypertensive participants showed significantly (p < 0.001) lower mean HGS (58.32 ± 14.19 N) and maximum HGS (215.26 ± 58.95 N) compared to normotensive controls (77.25 ± 20.64 N and 335.06 ± 113.22 N, respectively).

**Table 1:** Descriptive statistics of study participants. All data are represented as mean ± SD. SD: standard deviation; cm: centimeters; Kg: kilograms; BP: blood pressure; mmHg: millimeters of mercury; WHtR: waist-to-height ratio; N: Newtons; HGS: hand grip strength; HR: heart rate; BMI: body mass index; m^2^: square meter; S: significant; ES: extremely significant; NS: non-significant. All p-values reported are based on the Student’s t-test between the case and control groups. P-value < 0.05 is considered to be statistically significant.

Parameters	Controls (n = 50), Mean ± SD	Cases (n = 50), Mean ± SD	t statistic	p-value
Height (cm)	164.56 ± 7.61	162.53 ± 6.74	1.41	0.38 (NS)
Weight (kg)	59.31 ± 11.71	61.93 ± 7.37	1.34	0.43 (NS)
BMI (kg/m^2^)	24.03 ± 2.12	25.34 ± 4.21	1.76	0.080 (NS)
Waist circumference (cm)	81.22 ± 17.22	95.33 ± 19.03	3.89	0.016 (S)
WHtR	0.49 ± 0.10	0.587 ± 0.11	4.62	0.0067 (S)
Resting heart rate (HR)	78.03 ± 12.3	82.13 ± 13.42	1.59	0.0001 (ES)
Mean HGS (N)	77.25 ± 20.64	58.32 ± 14.19	5.35	0.0009 (ES)
Maximum HGS (N)	335.06 ± 113.22	215.26 ± 58.95	6.64	0.0004 (ES)
BP before exercise (mmHg)	116 ± 8.41	124 ± 18.27	2.81	0.049 (S)
BP after exercise (mmHg)	122 ± 11.39	132 ± 21.25	2.93	0.045 (S)

Pearson’s correlation analysis (Table 2) revealed a weaker negative association between WHtR and mean HGS (r = -0.19, p = 0.177), though not statistically significant. Similarly, WHtR and BP before exercise showed minimal correlation between them (r = 0.04, p = 0.778).

**Table 2:** Correlation between anthropometric parameters and hand grip strength. BP: blood pressure; WHtR: waist-to-height ratio; HGS: hand grip strength; HR: heart rate; S: significant; NS: non-significant. All r-values reported are based on Pearson's correlation test. P-value < 0.05 is considered to be statistically significant.

Variables	Correlation coefficient “r”	p-value
Waist circumference and BP before exercise	0.07	0.621 (NS)
WHtR and BP before exercise	0.04	0.778 (NS)
WHtR and max. HGS	-0.32	0.020 (S)
HR and max. HGS	0.28	0.044 (S)
WHtR and mean HGS	-0.19	0.177 (NS)

**Figure 1:**
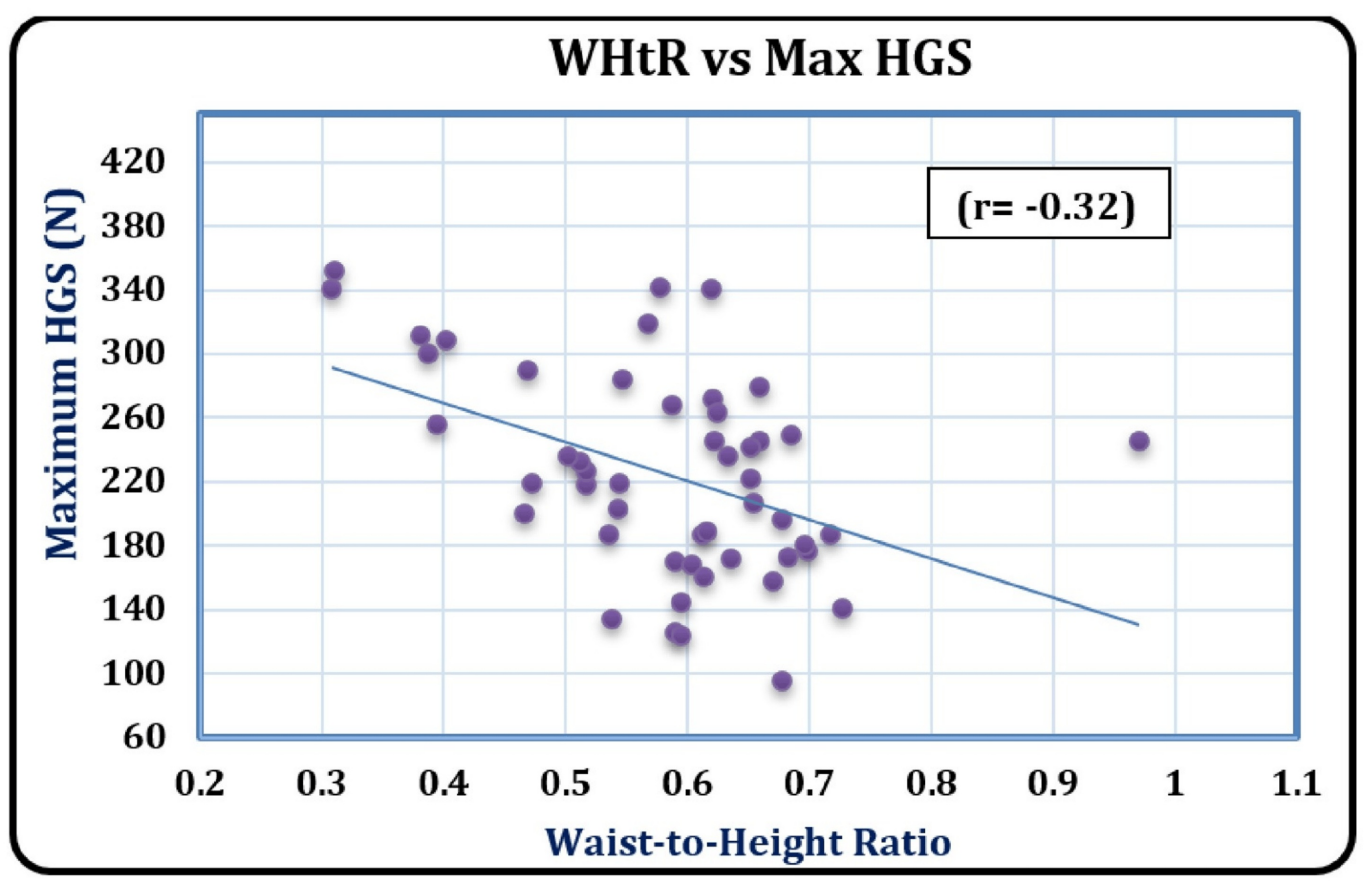
Scatterplot showing the correlation between waist-to-height ratio and maximum HGS among hypertensive cases. WHtR: waist-to-height ratio; Max: maximum; HGS: hand grip strength; r: Pearson's correlation coefficient.

As illustrated in Figure 1, a statistically significant, strong negative correlation (r = -0.32, p = 0.020) highlighted by the data analysis revealed the inverse relationship between maximum HGS and WHtR, whereby higher WHtR values are linked with lower grip strength.

**Figure 2:**
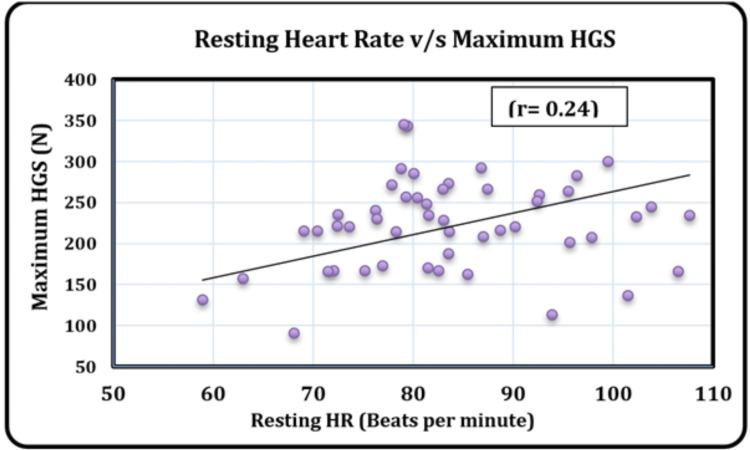
Scatterplot depicting the correlation between resting heart rate and maximum HGS among hypertensive cases. HR: heart rate; HGS: hand grip strength; r: Pearson's correlation coefficient.

The relationship between maximum HGS and HR was analyzed in cases, and as illustrated in Figure 2, it showed a statistically significant positive relation (r = 0.24, p = 0.044), implying that maximum grip strength increases with HR.

## Discussion

Hypertension is a predominant modifiable risk factor for cardiovascular outcomes, accounting for a substantial proportion of global mortality [7]. The present comparative cross-sectional study was designed to explore the association between body size parameters, handgrip strength, and hypertension in adults from rural central India.

The results of the study indicate decreased mean and maximum HGS of hypertensives in comparison to normotensive controls, pointing toward a potential relationship between muscular strength and cardiovascular health. This corroborates with the evidence [2,3,13,23], implying that diminished muscle strength is associated with an increased incidence of hypertension. It also supports the notion forwarded by earlier research that reduced muscle strength is linked to weakened vascular functions and metabolic imbalance [14,18,19].

Impaired strength in patients could probably be due to endothelial dysfunction, neuromuscular decline, or increased arterial stiffness, which are known contributors to elevated BP and cardiovascular morbidity and mortality [13,18,19,23]. These observations further potentiate the importance of muscular strength in maintaining overall cardiovascular integrity.

Improved relative muscle strength has a significantly lower risk of hypertension, highlighting the protective role of muscle function beyond mere muscle mass, as documented in a Korean study [24]. This study strengthened the link between HGS and hypertension and reported that people with better relative muscle strength had a significantly lower risk of hypertension.

Functional impairment of muscle has been linked to the presence of hypertension in past research [12] on patients presenting with reduced muscle quality owing to fat infiltration [25]. A study by Lee and Park [17] proposed a possible mediation of myosteatosis in contributing to hypertension and metabolic disorders in the Asian population.

The BP response to exercise in hypertensive subjects further supports the link between muscle strength and vascular function. Furthermore, the recorded increase in systolic BP after exercise among hypertensive patients was consistent with the prior hypothesis that the vascular resistance and autonomic dysfunction are more pronounced in those with lower muscle strength [13,18,19]. Prior studies indicated that improving muscle strength through isometric handgrip training can enhance vascular function and also can contribute to better BP regulation by potentially reducing BP over time, reinforcing the need for more strength-based interventions in hypertension management and control [5,8].

The exhibited association between HR and HGS was a non-linear one, where grip strength initially peaked with HR but later reduced after a point. Subjects having a modest HR depicted maximum HGS, while those at both the lowest and highest HR showed a weaker grip strength. This trend is in alignment with earlier studies [2,18,19,23] that showed optimal cardiovascular function leads to better muscular strength, but marked sympathetic overactivation (in tachycardiac states) may eventually reduce muscle performance due to greater muscle fatigue and vascular resistance.

The reduction in HGS at higher HR scores could also be attributed to disturbed hemodynamics and diminished oxygen delivery, which may reduce neuromuscular efficiency [3,5]. These findings are in agreement with the previous research that HGS is an index of overall cardiovascular health, with lower strength being linked to increased arterial stiffness and diminished cardiac output [15,25,26].

The association between WHtR and hypertension can be largely explained by the role of visceral adiposity in cardiovascular pathophysiology through mechanisms such as chronic low-grade inflammation and activation of the renin-angiotensin-aldosterone system, elevating the cardiovascular risk. Earlier studies have reported that greater WHtR is associated with higher hypertension risk, further supporting the association between metabolic dysfunction and reduced muscular strength [2,3,25]. In accordance with these observations, a significant difference in WHtR between hypertensive and normotensive subjects in this study also highlights the role of visceral fat owing to central adiposity in hypertension, emphasizing its influence on muscle function and strength decline. However, its correlation with grip strength in this study was weak, suggesting that while WHtR is associated with hypertension, its effect on muscle strength may be moderated by additional factors such as lifestyle habits and physical activity levels [25,27]. These findings suggest that while central adiposity is linked to hypertension, its direct impact on muscle grip strength remains inconclusive.

Patients with a higher WHtR demonstrated a dip in HGS, which aligns with current literature implying that excessive visceral fat can cause systemic inflammation, insulin resistance, and muscular atrophy, ultimately hampering HGS [18,19].

Additionally, it is important to recognize that HGS may be influenced by metabolic and systemic conditions, including diabetes and renal dysfunction, which could not be evaluated in the present study. Given the observational design of the study and the possibility of unmeasured confounding factors due to limitations in the availability of detailed clinical records, the results of the study should be interpreted cautiously.

Limitations

As the study was conducted on a relatively small sample, it becomes challenging to extrapolate the findings to wider populations. The study was conducted as a short-term project, so it may not have captured long-term trends or changes. The impact of lifestyle variables like dietary patterns and physical activity scores of study participants was not assessed, which might have influenced the observed associations. BP measurements in this study were obtained using clinic-based readings rather than home or ambulatory BP monitoring, which may provide a more comprehensive BP assessment according to contemporary hypertension guidelines.

Some medical and metabolic conditions known to influence HGS, such as diabetes mellitus, chronic kidney disease, previous major adverse cardiovascular events, and metabolic disorders through mechanisms including metabolic dysregulation, systemic inflammation, and muscle wasting, were not systematically evaluated or compared between the study groups. Due to the study design and the availability of clinical data in the rural setting where the study was conducted, complete documentation of all metabolic comorbidities was not available for all participants at the time of data collection, and they could not be used for matching between groups. Information regarding the number of antihypertensive medications was also not consistently documented, which limited the ability to assess hypertension severity or treatment burden, so stratification by hypertension stage was not feasible. Hence, the study could not differentiate between types or grades of hypertension, which potentially may have impacted the results.

The authors acknowledge that all these factors could potentially confound the observed associations, so the findings should be analyzed with appropriate caution. Owing to the absence of detailed evaluation of confounders (such as metabolic comorbidities or medication burden), the ability to establish a definitive causal relationship remains limited.

Future directions

Further research with larger cohorts and comprehensive assessments, including biochemical markers such as glycosylated hemoglobin and renal function indicators like estimated glomerular filtration rate, with detailed medication profiles of hypertension, along with evaluation of metabolic diseases such as diabetes and renal dysfunction, is warranted to better understand their potential influence on HGS and cardiovascular risk. Future studies with longitudinal designs are required to further explore the causal relationships between muscle strength, body composition, and hypertension.

## Conclusions

The study provides insights into the potential relationship between hypertension, body size parameters, and muscle grip strength. The hypertensive patients of the study demonstrated significantly reduced mean and maximum HGS compared with normotensive participants, pointing toward the role of muscle function and the potential utility of HGS assessment as a simple, practical, yet effective screening tool for cardiovascular risk evaluation, particularly in rural and resource-limited settings. Both anthropometric measures, i.e., waist circumference and WHtR, were significantly higher in the hypertensive group. Maximum HGS in hypertensives revealed a statistically significant negative association with WHtR, underpinning its role as a crucial metric for identifying elevated cardio-metabolic risk tied to central adiposity. However, further longitudinal studies incorporating larger cohorts and comprehensive adjustment of confounders are required to better understand the causal relationships between the variables.
